# Nanomaterials Electrochemistry: Insights for Materials Scientists and Electrochemists

**DOI:** 10.1021/acsnano.6c08511

**Published:** 2026-08-04

**Authors:** Mario Urso, Mahnaz Azimzadeh Sani, Richard G. Compton, Kristina Tschulik, Martin Pumera

**Affiliations:** † Dipartimento di Fisica e Astronomia “Ettore Majorana, Università degli Studi di Catania, Catania 95123, Italy; ‡ CNR-IMM, Catania 95123, Italy; § Analytical Chemistry, Faculty of Chemistry and Biochemistry, Ruhr University Bochum, Bochum 44780, Germany; ∥ Physical and Theoretical Chemistry Laboratory, 6396 University of Oxford, South Parks Road, OX1 3QZ Oxford, Great Britain; ⊥ Future Energy and Innovation Laboratory, Central European Institute of Technology, Brno University of Technology, Purkynova 123, 61200 Brno, Czech Republic; # Department of Medical Research, China Medical University Hospital, China Medical University, No. 91 Hsueh-Shih Road, Taichung 40402, Taiwan; ∇ Office of Research and Development, Asia University, Taichung 40402, Taiwan; ○ Energy Research Institute@NTU (ERI@N), Research Techno Plaza, X-Frontier Block, Level 5, 50 Nanyang Drive, 637553 Singapore, Singapore; ◆ Advanced Nanorobots & Multiscale Robotics Laboratory, Faculty of Electrical Engineering and Computer Science, VSB-Technical University of Ostrava, 17. Listopadu 2172/15, 70800 Ostrava, Czech Republic

**Keywords:** nanomaterial transformation, electrocatalysis, structure−activity relationships, impurities, *operando* characterization, electrode architecture, mass transport, reproducibility in electrochemistry

## Abstract

Electrochemistry is a key enabler of modern technology, underpinning materials processing, chemical synthesis, and energy storage and conversion. Electrochemistry requires electrodes—conducting materials to external circuit. However, the actual composition of these electrodes is often far removed from that assumed in electrochemical equations—atomically flat homogeneous surfaces. Electrochemical response of nanomaterials arises from a complex interplay of composition, morphology, structure, defects, and surface properties, coupled with mass transport. Failing to fully account for this complexity can lead to ambiguous interpretations of electrochemical activity and reaction mechanisms, particularly at the nanoscale where apparent activity may reflect measurement and transport artifacts rather than intrinsic material properties. This perspective critically examines common sources of ambiguity that emerge when studying nanostructured and heterogeneous materials in electrochemical environments. Drawing on insights from both materials science and electrochemistry, we highlight how factors such as nanomaterial transformation, impurities, electrode architecture, and transport limitations can complicate mechanistic understanding and hinder reliable comparison across the literature. Particular emphasis is placed on the need for rigorous nanomaterial characterization, careful experimental design, and transparent reporting practices, especially in the context of increasingly data-driven research. At the same time, we show that classical electrochemical models, which assume well-defined and homogeneous molecular systems, are not directly transferable to real, complex nanostructured electrode materials. By outlining practical considerations and conceptual pitfalls, this work aims to support more reliable, reproducible, and meaningful investigations at the crossroad of materials science and electrochemistry, highlighting how the nanoscale introduces challenges that require particularly careful experimental design and interpretation. We hope that electrochemists and materials scientists will learn from each other to move this key field forward.

## Graphical abstract

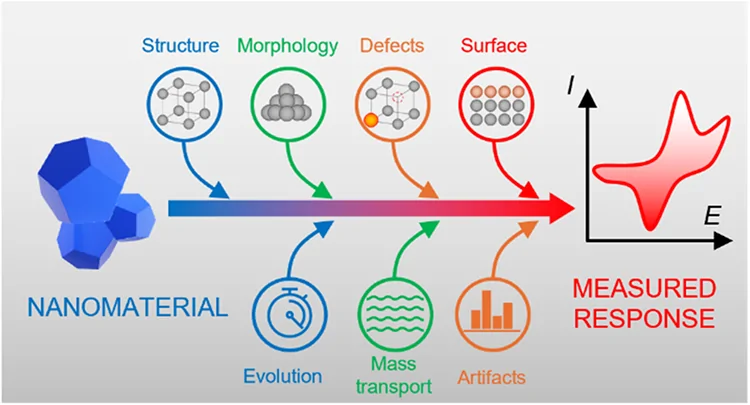

## 1 Introduction

Electrochemistry and materials science have become increasingly connected to the point that progress in one field is often subject to advances in the other. Electrochemical processes support a wide range of technologies, from energy conversion and storage (batteries, fuel cells, electrolyzers) to sensing, environmental remediation, and synthesis.^1–8^ In nearly all these applications, the performance is ultimately governed not exclusively by molecular (electro-)­chemistry but additionally and often critically by the properties of electrode materials, typically complex, nanostructured, and nonstatic under the operating conditions.

At the same time, this convergence introduces substantial challenges at the nanoscale, where classical electrochemical assumptions begin to break down. Electrochemistry is traditionally rooted in frameworks developed for well-defined chemical and electrode systems, where species, concentrations, and reaction pathways are clearly identifiable. By contrast, practical nanoscale or large-scale materials are intrinsically heterogeneous and evolve under electrochemical conditions.^9^ The physicochemical properties of nanomaterials, such as phase, morphology, structure, surface chemistry, defects, and composition, crucially influence the electrochemical behavior, while measured responses reflect a convolution of mass transport and kinetic effects, both homogeneous and heterogeneous.^10–13^ As a result, the direct application of classical electrochemical models can lead to oversimplified interpretations, while insufficient nanomaterial characterization can obscure the true origin of observed activity.

These issues are further amplified by the increasing use of complex data sets and data-driven approaches, where the quality and consistency of input parameters critically determine the reliability of the output.^14,15^ Inadequate description of nanomaterials, incomplete reporting, neglect of experimental artifacts, and methodological errors can therefore propagate into misleading conclusions, limiting reproducibility and comparability across studies.^16^

In this perspective, we write as both materials scientists and electrochemists, drawing on our combined experience at the interface of these disciplines. Rather than providing a comprehensive review, we aim to offer a set of practical considerations and critical reflections, structured as mutual advice, highlighting common difficulties and overlooked aspects in the study of nanoscale materials for electrochemistry. By explicitly addressing the assumptions, limitations, and methodological gaps that arise when bridging these fields, we hope to contribute to more rigorous, transparent, and meaningful investigations.

## 2 Advice to Electrochemists from Materials Scientists

### 2.1 Nanomaterials Are Not Molecules

Electrochemistry is traditionally formulated in terms of well-defined chemical species, reaction pathways, and thermodynamic or kinetic parameters. For molecular redox couples in solution, such as Ru­(II)/Ru­(III) hexaamine, the structure, bonding characteristics (including energies, strengths, and distances), and redox behavior are well established, and the measured electrochemical response can be directly related to known physicochemical parameters under controlled conditions. Such homogeneous molecular systems contrast with heterogeneous electrode ones, where surface structure, active sites, and adsorption energetics are often not uniquely defined, leading to a higher complexity.^13^ Even for nominally simple redox couples (*e.g.*, ferricyanide/ferrocyanide), deviations from pure outer-sphere electron transfer kinetics can arise depending on the electrode surface and history, such as pre-exposure to organic solvents.^17^ Therefore, the conceptual simplicity breaks down when moving from molecular or solution-phase chemistry to solid materials, especially in nanoscale structures. When an electrode material is described as “Pt”, for instance, this designation does not correspond to a single, well-defined system, but rather to a broad class of nanomaterials that can differ in phase, morphology, structure, surface chemistry, defects, batch variability, and processing history.^18^ Therefore, the measured electrochemical response should be better understood as a property of a specific material state, influenced by multiple, interdependent variables. Figure 1 schematically illustrates this concept.

**Figure 1:**
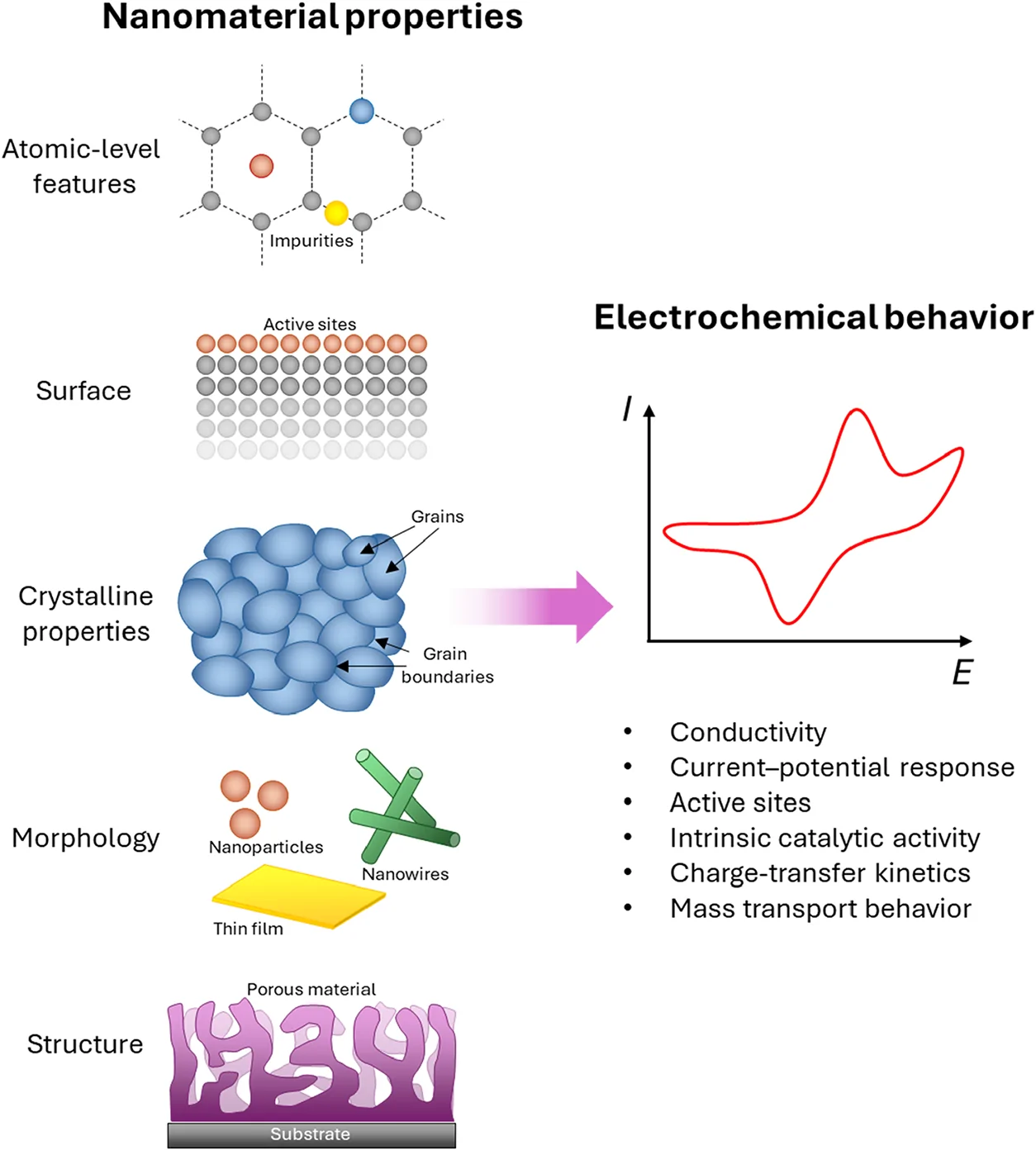
Multiscale origin of electrochemical response in nanomaterial-based electrodes. The electrochemical response of nanomaterials results from phenomena across multiple length scales: atomic-level features, such as impurities, and electronic structure define the intrinsic reactivity, while nanoscale morphology, phase, and interfaces control the accessibility of active sites and charge transport. At larger scales, porosity governs mass transport.

Although molecular systems can also display structure-dependent reactivity, these variations are typically discrete and well-defined. However, at the most basic level, the stoichiometry does not uniquely define the electrochemical behavior of a material. Different phases, such as crystalline polymorphs, amorphous states, or multiphase mixtures, may exist at the identical nominal stoichiometry and still exhibit markedly different electrochemical properties due to variations in mass and charge transport. This is exemplified by (i) polymorphic systems such as V_2_O_5_, where metastable crystal structures exhibit markedly different ion diffusion pathways and electrochemical capacities compared to their thermodynamically stable counterparts;^19^ (ii) amorphous NiFe catalysts, where short-range order and structural disorder enable the exposure of a larger number of electrochemically active sites and enhance water oxidation activity compared to crystalline analogues;^20^ (iii) and multiphase systems such as WO_3_, where the formation of hexagonal/monoclinic phase junctions significantly improves hydrogen evolution activity compared to single-phase nanomaterials.^21^ Moreover, electrochemical operation itself can, and frequently does, induce phase transformations. In this sense, electrochemical measurements often probe an evolving material whose phase can partially or fully change under the applied potential or current.^22^

Beyond the phase, nanomaterial morphology and microstructure introduce additional layers of complexity. Parameters such as particle size, porosity, grain boundaries, and interparticle connectivity directly influence charge distribution, conductivity, and the accessibility of active sites. For instance, the distribution and connectivity of pores can control ion transport pathways and local concentration gradients, with hierarchical porosity often enabling improved rate capability by balancing ion accessibility and storage.^23^ Similarly, particle morphology and crystallinity can profoundly affect the electrochemical behavior: nanostructured or porous particles may enhance surface area and shorten diffusion lengths,^24^ whereas single-crystal materials can exhibit improved conductivity compared to their polycrystalline counterparts due to the absence of grain boundaries and reduced defect accumulation.^25^ Also, interfaces play an important role, as their structure and chemistry govern charge transfer and interfacial resistance. Variations in surface roughness, wettability, or phase connectivity can lead to nonuniform current distributions and localized reaction environments, particularly in heterogeneous or composite electrodes.^26^ In polymeric and carbon-based systems, microstructural organization, such as phase segregation, crystallinity, or the formation of interconnected transport pathways, can strongly influence ionic and electronic conductivity, as well as long-term stability.^27,28^ As a result, two materials with identical composition and phase may display substantially different electrochemical responses if their morphological and structural properties differ. The electrochemical signal thus reflects not only intrinsic reaction kinetics, but also the spatial organization and hierarchical architecture of the material in which the reaction occurs. This is exemplified by carbon-based electrodes, where allotropes such as graphite, graphene, carbon nanotubes, and diamond exhibit different electrochemical properties associated with their distinct electronic structures and bonding configurations.^29^ It has been shown that the basal plane of sp^2^ carbon is much less electrochemically active than edges.^30^ Graphene offers high surface area and efficient charge transport in two-dimensional architectures, carbon nanotubes provide highly conductive and high-aspect-ratio networks that facilitate electrical wiring of redox-active species, and diamond is characterized by an exceptional chemical stability.^31,32^

Surface chemistry further complicates this picture. Electrochemical processes are inherently interfacial, occurring at the boundary between the electrode and the electrolyte. However, the surface of a nanomaterial is rarely a simple truncation of the bulk structure. Instead, it may exhibit reconstruction, segregation, adsorption of species from the electrolyte, or the formation of passivation layers. In addition, surface terminations and chemical functionalization can strongly modulate electronic structure, wettability, and interfacial reactivity, as widely observed in systems such as carbon-based nanomaterials and MXenes, where specific surface groups directly influence charge storage, redox behavior, and catalytic activity.^33–35^ Consequently, the surface composition and electronic structure, which are the most directly relevant to electrochemical reactions, can differ significantly from bulk properties. Hence, modifications at the surface, whether intentional or incidental, may dominate the observed electrochemical behavior, further highlighting the necessity to distinguish between bulk and interfacial characteristics by proper surface characterization.

An additional factor is the presence of defects, impurities, and disorder, which have been traditionally considered detrimental. Real materials invariably deviate from ideal crystalline order, containing point defects such as vacancies and interstitials, line defects like dislocations, planar defects such as grain boundaries, and other defects. These defects are not merely perturbations but often fundamentally determine material properties, including ionic and electronic conductivity, catalytic activity, and chemical stability. For example, in carbon-based and transition-metal-based nanomaterials, point defects like vacancies and heteroatom doping can redistribute electronic density, create high-energy active sites, and lower ion migration barriers, thereby enhancing electrochemical performance.^36,37^ Similarly, in metal oxide electrocatalysts, anion vacancies have been shown to modulate the adsorption energetics of key intermediates and accelerate reaction kinetics.^38^ In electrocatalysis, defects therefore can not only serve as active sites but also as transport pathways, meaning that their concentration and distribution directly influence the catalytic activity.^39^ In Section 2.3, a detailed discussion of the effects of impurities is provided. In addition to these effects, the crystallographic orientation of the surface of a material can also critically influence its electrocatalytic activity. For instance, surface reconstruction phenomena and facet-dependent activity have been reported in Co- and Ir-based electrocatalysts.^40,41^ As highlighted in recent analyses of battery nanomaterials, understanding and controlling defects and impurities is considered the key to achieving better energy storage performances.^42^ However, defect-related activity should not be overinterpreted. Indeed, in this regard, a recent study employing scanning electrochemical cell microscopy revealed that the apparent enhancement in electrocatalytic performance originates from measurement artifacts rather than Pt grain boundaries.^43^

Finally, materials are inherently subject to variability arising from synthesis, processing, and operation. Differences in precursor purity, synthesis route, postsynthesis processes (*e*.*g*., thermal and chemical treatments, electrode fabrication method), storage conditions, and operational errors can produce nanomaterials with distinct combinations of phase, morphology, and defect structure, even when nominally identical protocols are followed. This variability is a major contributor to the reproducibility challenges widely reported in electrochemistry.^16,44,45^ Measurements that appear inconsistent or contradictory may, in fact, reflect unrecognized differences in the underlying materials rather than fundamental discrepancies in electrochemical mechanisms.

The above considerations highlight a central point that must be clear to all electrochemists approaching materials science: electrochemical techniques, such as cyclic voltammetry (CV) or electrochemical impedance spectroscopy (EIS),^46^ yield signals that convolute contributions from phase, microstructure, morphology, surface chemistry, and defects or impurities. Without explicit consideration of these factors, the interpretation of electrochemical data remains ambiguous, and comparisons between different materials or studies become difficult and unreliable. Therefore, recognizing that materials, specifically nanomaterials, are not atomically precise chemicals is a necessary step toward a more rigorous and reproducible electrochemistry, in which the observed electrochemical behavior is consistently linked to well-defined physicochemical properties rather than nominal compositions alone. To minimize ambiguity, authors should report a material using two descriptors whenever possible: (i) the nominal stoichiometry arising from synthesis (*e.g.*, V_2_O_5_, NiFeOx), and (ii) the experimentally (*in situ*/*operando*) characterized surface state that is actually exposed to the electrolyte, including surface composition, oxidation states, functionalization, and detectable impurities.

### 2.2 Rigorous Nanomaterials Characterization Is a Prerequisite for Reliable Electrochemistry

A central requirement when using materials, especially nanostructured ones, in electrochemistry is the need for rigorous and explicit material characterization. Since the electrochemical response of a material reflects a convolution of phase, morphology, structure, surface chemistry, and defects, incomplete or poorly reported properties directly translate into ambiguity in interpretation and limited reproducibility of the electrochemical behavior. In this context, characterization is not a supplementary step, but a prerequisite for establishing meaningful structure–property relationships: any claim regarding the electrochemical activity, selectivity, or mechanism is only as reliable as the description of the material on which it is based.

In practice, this implies that no single technique is sufficient to define a material, and that a comprehensive combination of complementary methods is required to capture its properties from multiple, interconnected perspectives. At a minimum, a consistent description requires information on phase, morphology, composition, and surface chemistry, typically obtained through X-ray diffraction (XRD), electron microscopy (SEM/TEM), often coupled with energy-dispersive X-ray spectroscopy (EDX), and surface-sensitive techniques such as X-ray photoelectron spectroscopy (XPS). Each method captures only a partial aspect of the material: XRD provides information on crystalline phases but may overlook amorphous components; SEM and TEM reveal morphology and local structure but may probe limited regions; EDX offers elemental analysis with detection limits typically around 0.1 wt % depending on the element and experimental conditions;^47^ and XPS is intrinsically surface-sensitive, probing only a few nanometers in depth with elemental detection limits around 0.01–10 at %, depending on the element and matrix.^48^ In addition, spectroscopic methods, including vibrational techniques such as Raman and Fourier-transform infrared (FTIR) spectroscopy, as well as UV–vis spectroscopy and photoluminescence, provide critical insight into bonding, local structure, and energy band diagram, which are often directly relevant to electrochemical and photoelectrochemical behaviors. Of note, all these methods provide valuable complementary information, but each has intrinsic limitations in sensitivity, spatial or depth resolution, and sampling volume. However, (quasi)­reversible transformations of the material under electrochemical operation may still remain hidden, unless suitable *operando* characterization can be conducted.

The importance of material characterization is clearly illustrated in systems where structural claims are intrinsically difficult to verify. A notable example is provided by single-atom catalysts (SACs), where the electrochemical activity is attributed to isolated metal atoms dispersed on a support.^49^ Demonstrating the absence of clusters or nanoparticles in these systems requires a combination of advanced techniques, including aberration-corrected TEM, X-ray absorption spectroscopy (XAS), and surface-sensitive probes. Early studies highlighted how incomplete characterization could lead to overinterpretation of atomic dispersion, prompting the field to adopt increasingly stringent, multitechnique validation protocols.^50^ This case exemplifies a broader principle: the more specific the claim, the more demanding the required characterization.

Equally instructive are examples in which insufficient characterization leads to fundamentally misleading conclusions. In oxygen evolution reaction (OER) studies, it is now well established that Fe impurities, introduced from electrolytes, glassware, or electrode preparation, can dramatically enhance the apparent activity of Ni- and Co-based catalysts.^51^ In particular, careful investigations have shown that catalytic performance initially attributed to intrinsic nanomaterial properties can, in fact, originate from Fe incorporation at trace levels, which may remain undetected without targeted analysis.^52,53^ Similarly, in hydrogen evolution reaction (HER) measurements, Pt contamination from counter electrodes can lead to significant overestimation of activity in nominally nonprecious systems.^54^ In both cases, the electrochemical signal is dominated by species that are not captured by nominal composition, illustrating how minor or unintended components can control macroscopic behavior.

Even when contamination is not a factor, insufficient characterization can introduce substantial quantitative uncertainty. For example, electrochemically active surface area (ECSA), which is frequently used to normalize catalytic activity, is often treated as a proxy for the real active surface area of a nanomaterial, although its determination relies on indirect electrochemical measurements and model-dependent assumptions.^55^ Without independent structural and surface characterization, such estimates can be unreliable, leading to potentially misleading comparisons.

These challenges are reflected in broader concerns about reproducibility in electrochemistry: systematic analyses have shown that electrochemical measurements are highly sensitive to both experimental conditions (including temperature, leakage from or potential drift of reference electrodes) and poorly characterized material properties, potentially leading to discrepancies that are incorrectly interpreted as mechanistic differences.^16^

At the same time, the application of artificial intelligence (AI) and machine learning (ML) in nanomaterial electrochemistry requires high-quality data sets that enable explicit correlations between measured material properties and functional performance. Recent reviews have highlighted the growing role of AI-driven and ML approaches in the discovery of nanomaterials and their electrochemical analysis, while also emphasizing their dependence on robust and reliable data foundations.^14,15,56^ However, if the underlying materials are poorly characterized or inconsistently described, the resulting data sets inherently include hidden variability and bias, which ultimately propagate through the models and affects the reliability of the results. As a consequence, the predictive performance, generalizability, and interpretability of these approaches are fundamentally limited by the quality, completeness, and consistency of the input data, rather than by the algorithms themselves. This highlights the critical need for rigorous and standardized nanomaterial characterization as a prerequisite for meaningful data-driven modeling. Arguably, at present, it is the sheer variation and unreliability of much electrochemical data which are handicapping the application of AI/ML in electrochemistry. For example, the reported standard electrochemical rate constant for the ferrocene/ferrocenium redox couple in acetonitrile spans more than 2 orders of magnitude, while diffusion coefficient data is notoriously unreliable, partly due to neglect of basic thermostatting at the point of measurement and often from the misanalysis of voltammetric data such as peak current/scan rate data.

These considerations establish a clear requirement for scientific manuscripts on nanomaterial-based electrochemistry: electrochemical measurements must be accompanied by an equally rigorous and transparent description of the nanostructures. Without such information, electrochemical data remain difficult to interpret and impossible to generalize. Conversely, when supported by comprehensive characterization, electrochemical measurements can be meaningfully connected to underlying nanomaterial properties, enabling reproducibility, comparison across studies, and the development of predictive understanding.

### 2.3 Role of Impurities

Most electrocatalyst materials are neither tested nor applied as ideal, flat electrodes but as composite, porous films, which are nanoparticles mixed with carbon and ionomer, deposited on foils, meshes, or gas-diffusion layers. In such systems, mass transport is no longer a simple semi-infinite diffusion problem, and the intuition built on smooth macroelectrodes or isolated nanoparticles breaks down. Porosity, tortuosity, film architecture, and the distinction between isolated particles and interacting ensembles can all transform the observed electrochemical response, sometimes in ways that mimic changes in intrinsic activity.

Consider an electrode with its surface modified by particles which act as electrocatalysts for a reaction of choice. Clearly, at zero loading, no (catalytic) current will flow, while at high loadings (and at suitably high applied potentials) large currents are observed that are controlled by diffusion to the entire electrode (substrate) as a whole and hence become effectively independent of the catalyst loading. For isolated nanoparticles sitting on an inert support, the steady-state diffusion field around each particle is essentially hemispherical. The smaller the particle, the higher the steady-state flux per geometric area, which is the familiar basis of nanoelectrochemistry. At sufficiently low, submonolayer coverages with large interparticle separations and experiments conducted on a time scale such that the diffusion layers associated with the particles do not overlap (“diffusional independence”), the response is simply that of the sum of the isolated particles. However, as coverage increases (or in a voltammetric experiment, the scan rate decreases) while maintaining a submonolayer coverage of particles, the diffusion layers of separate particles begin to overlap, and the system transitions from isolated to ensemble behavior. Thus, at these intermediate loadings, the response depends on the scan rate (in the case of voltammetric measurements), the particle size and the average particle-to-particle separation distance. The current increases sublinearly with the number of particles until, at high coverages, the overall response resembles that of a fully active macroelectrode with a shared, planar diffusion layer, and the characteristic radial diffusion at single nanoparticles disappears. Therefore, the transition to full diffusion control is realized, reflecting a regime in which the maximum turnover of the reagents is ultimately limited by the rate of diffusion to the geometrical area of the substrate electrode. These mass transport contributions can be readily accounted for by numerical simulations, to distinguish true “nano” size effects on charge transfer kinetics from mere geometric ones causing increased current densities at nanostructured electrodes.^57,58^ Figure 2 shows how the diffusion layers—corresponding to reactant depletion—around individual particles overlap with increasing coverage, ultimately forming a large, uniform diffusion layer over the entire electrode (substrate) surface. It follows from the strongly nonlinear dependency of the catalytic current on the catalyst loading that data should be reported alongside the coverage of the catalyst in terms of g m^–2^ and not, for example, on a per gram basis and include particle size data.

**Figure 2:**
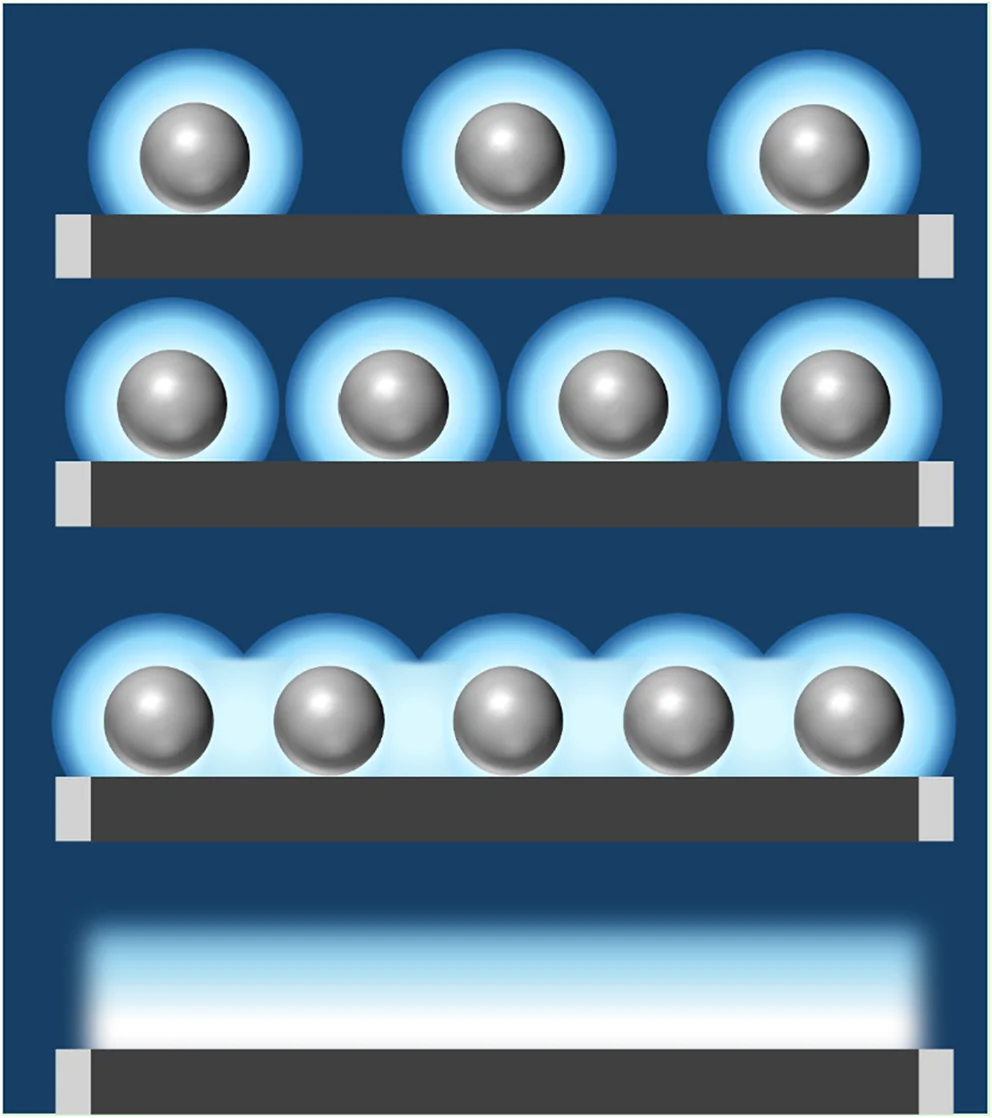
Schematic representation of diffusion fields around catalyst particles from isolated to ensemble and macroelectrode behavior.

The ability of a small coverage of electrocatalyst particles on an electrode surface, together with the application of a suitable potential to drive currents at or near the levels associated with diffusion-controlled reaction at a level corresponding to the geometrical area of the substrate electrode, has significant implications with respect to the essential need for the experimenter to be wary of possible impurities in the sample of electrocatalyst particles. Even a tiny amount of impurity particles, which themselves are more strongly catalytic than the majority species, can show an electrochemical response which dominates the voltammetry. This is because as the potential is swept either anodically or cathodically, as appropriate for the process of interest, reaction is induced to occur first at the more catalytic particles, leading to reactant depletion, so that when the voltammetric scan reaches the larger potentials required for the less catalytic but majority particles to induce electrolysis, the amount of reactant near the electrode surface has been substantially depleted and so little signal is seen corresponding to these particles, since there is no reactant remaining to undergo catalyzed reaction. Thus, the voltammetry reveals strong electrocatalysis but at a potential reflecting the impurity (minority) particles, not the sample under investigation. This is not a hypothetical problem but rather one that is all too commonly encountered. Historically, the problem was first recognized in respect of the claimed electrocatalytic properties of multiwalled carbon nanotubes, where the observed catalysis with respect to diverse electrode processes, most notably the oxidation of hydrazine in aqueous solution, was shown to be due to the presence of metal oxide particles (notably Fe_2_O_3_) resulting from the synthesis of the nanotubes by chemical vapor deposition.^59^ Notably, the attempted purification of the nanotubes (already at >95% purity) *via* “super-washing” was shown to be ineffective at removing the residual iron oxide particles. Subsequent work showed similar effects caused by residual trace iron oxide in respect of the electro-reduction of hydrogen peroxide^60^ and trace copper oxide in respect of the electroanalytical detection of glucose using multiwalled carbon nanotubes.^61^ Copper-based impurities were also inferred to cause apparent—but false—nanotube electrocatalysis in the detection of the anesthetic gas, halothane.^62^ Work also showed that “super-washing” was ineffective in purifying single-walled carbon nanotubes.^63^ For some materials, notably single- and multiwalled carbon nanotubes, complete removal of residual metallic and metallic oxide impurities may not be achievable by postsynthetic washing. In such cases, the identity and concentration of impurities should be explicitly reported, and their contribution to the electrochemical response should be carefully investigated.

Similarly, several studies showed that the chemical synthesis of graphene inherently introduces metallic impurities that alter its electrochemical behavior.^64–66^

The clear implication of the above is that experimenters need to be vigilant with respect to the characterization and analysis of their materials when using them for electrochemistry. While the presence of impurities at the ca. 1% level is usually adequate for conventional synthetic works, in electrochemistry, much higher demands are made if electrocatalysis is assumed to be revealed by the lowering of potentials required to bring about a sought transformation.

### 2.4 Nanomaterials Are Dynamic under Operating Conditions

A further complication, which is often underestimated in electrochemical studies, is that nanomaterials, and in general materials, are not static during operation. Typically, they are characterized before the electrochemical measurements and only rarely after electrochemical measurements. Moreover, these characterizations are performed *ex situ*, under vacuum or ambient conditions, while the actual electrochemical environment involves applying a potential, interactions with an electrolyte, and a continuous flux of ions and electrons. Under these conditions, nanomaterials can undergo substantial and irreversible changes in composition, morphology, structure, and surface chemistry. As a result, the nanomaterial being characterized before the measurement may differ significantly from the nanomaterial responsible for the observed electrochemical response (Figure 3).

**Figure 3:**
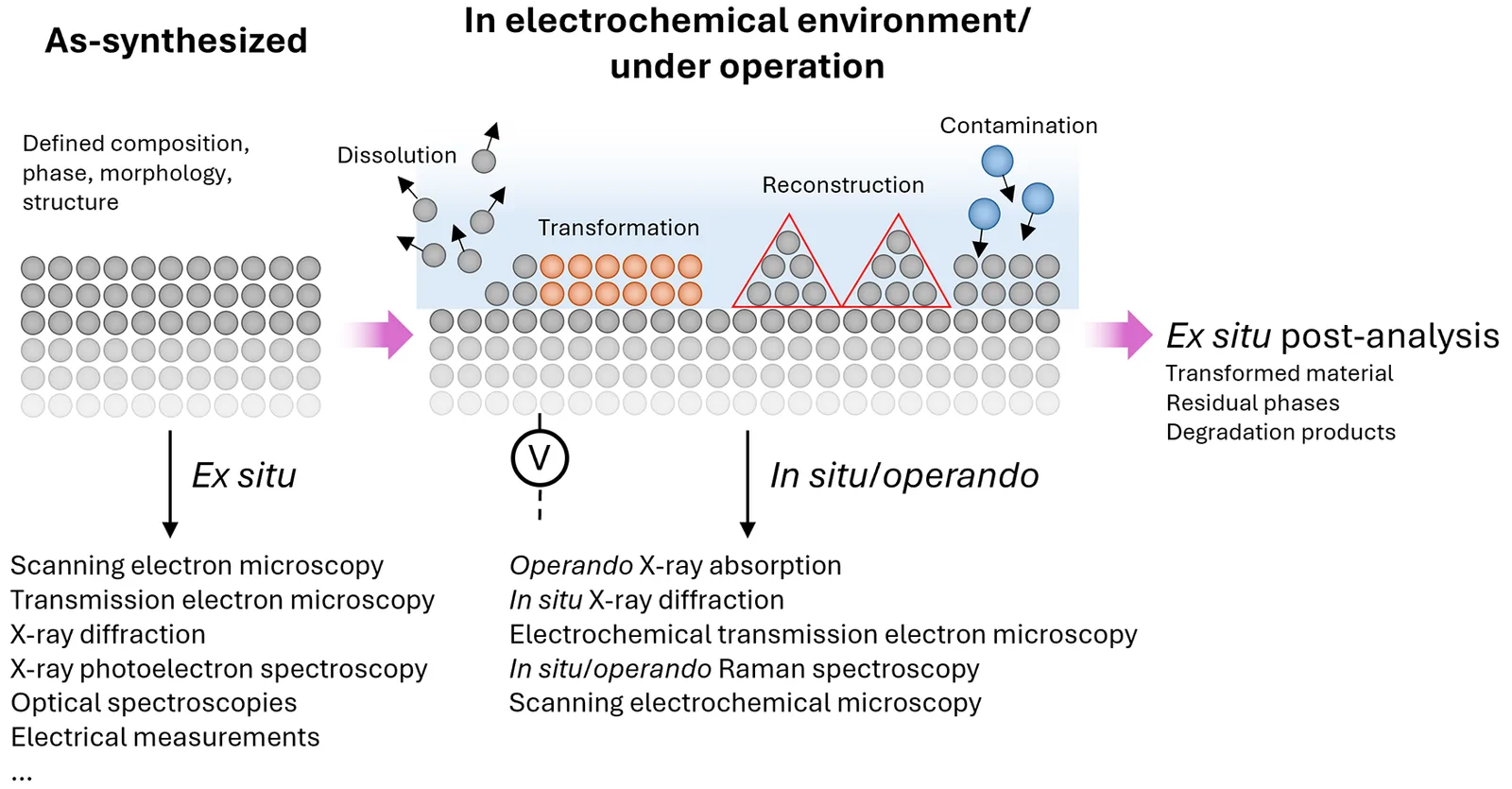
Characterization follows the nanomaterial across its lifecycle, from the as-synthesized state to the active state under operation and the postmeasurement state. Each stage provides distinct information, and only their combination enables a correct and comprehensive interpretation of the electrochemical behavior.

In this context, one of the most common processes is the dissolution or corrosion of the active material. Transition metal oxides, sulfides, and phosphides, and also nominally stable catalysts, can partially dissolve under operating conditions, especially in strongly acidic or alkaline electrolytes.^67,68^ Subsequently, the dissolved species may redeposit elsewhere, on the electrode surface or within the cell, leading to uncontrolled contamination and unpredictable and unreproducible results.

Another recurring phenomenon is the formation of a different active phase during electrochemical testing. This circumstance is particularly relevant in electrocatalysis, where the active phase may not correspond to that of the as-synthesized nanomaterial, but rather to a dynamically formed surface layer or reconstructed phase. For instance, in OER, many transition metals undergo surface transformations into oxyhydroxide phases, which are now widely recognized as the true active species in systems initially described as metals, oxides, or hydroxides.^69^ This is the case of Ni, NiO, and Ni­(OH)_2_ nanostructures, which, when processed in alkaline media, form a thin NiOOH surface layer, which is then responsible for the OER electrocatalytic activity,^70–72^ and is equally true for Co-based OER catalysts.^41,73^

Morphological and structural changes also play an important role. The evolution of gas bubbles, volume changes during ion insertion, or weak adhesion of the material on the substrate can lead to its detachment or the formation of cracks.^74^ These processes can increase the effective surface area, eventually improving ion diffusion, but modify the connectivity of the material, thereby influencing its electrical conductivity and, in turn, electrochemical performance.^75,76^ In some cases, the measured electrochemical response may reflect not only intrinsic material properties but also the progressive degradation of active material during operation and the exposure of the substrate.

Apart from the loss of electrode material, restructuring can occur during the electrochemical experiment. Once again, SACs provide an illustrative example: despite these systems being defined by the presence of isolated metal atoms, electrochemical conditions can induce migration and aggregation of these atoms, forming clusters or nanoparticles.^77^ This dynamic behavior complicates the identification of the active site and the stability of the electrochemical performance, highlighting the necessity of monitoring structural evolution under working conditions rather than relying solely on the initial characterization.

Even if the overall morphology or structure of a nanomaterial appears preserved, slight changes in average oxidation state or local coordination can significantly impact its electrochemical behavior. Surface poisoning and adsorption phenomena further complicate this picture.^78^ Electrolyte species, impurities, or reaction intermediates can adsorb on the nanomaterial surface, blocking active sites or altering reaction pathways.^79,80^ These effects are typically potential-dependent and may evolve over time, leading to incorrect evaluation of the material activity and unstable or unreproducible electrochemical signals. An instructive parallel can be drawn with electrochemical sensing, where the detection principle itself relies on the sensitivity of the material to interfacial changes induced by the adsorption or reaction with the analyte.^81^ In many sensing platforms, the analyte modifies the local electronic structure, surface charge distribution, or interfacial kinetics of the material, leading to changes in the measured current, potential, or impedance. These responses are, then, interpreted as signals rather than perturbations, but they arise from the same fundamental mechanisms discussed above, *i.e.* the dependence of the electrochemical behavior on the evolving state of the material at the interface. From this perspective, electrochemical sensing can be considered as a deliberate exploitation of material sensitivity to interfacial chemistry.

These considerations emphasize that nanomaterials must be characterized also after the electrochemical experiments. Comparing the material before and after the tests not only provides essential information on its stability and degradation, but also, in many cases, only such a comparative analysis can reveal whether the observed electrochemical behavior arises from the intended nanomaterial, a transformed surface layer, or a degradation product.^78^

Importantly, postmeasurement characterization alone can be insufficient to fully capture the dynamic nature of electrode materials. Many of the previously described transformations, such as phase reconstruction, dissolution/redeposition, or changes in oxidation state, can occur transiently and may not be preserved once the applied potential is removed or the system is not in contact with the electrolyte and returned to ambient conditions. As a result, *ex situ* analysis may provide only a partial or even misleading picture of the active material. This limitation has driven the increasing utilization of *operando* and *in situ* characterization techniques, which enable direct observation of materials under working electrochemical conditions.^82,83^ Specifically, techniques such as *operando* XAS, *in situ* XRD, and electrochemical transmission electron microscopy (EC-TEM), as well as electrochemical Raman spectroscopy (including surface-enhanced Raman spectroscopy)^84,85^ and dark-field microscopy,^86^ allow tracking the oxidation states, phase evolution, and structural changes in real time, under applied potential, and in the presence of the electrolyte. Notably, Raman-based approaches and dark-field microscopy offer more accessible alternatives to synchrotron-based techniques, such as XAS. For example, these approaches have been crucial for monitoring the evolution of coordination environments in SACs and in revealing dynamic restructuring processes that may be otherwise invisible *ex situ*.^87^ In addition, the use of this time-resolved characterization is vital for the formulation of a reliable reaction mechanism: without knowledge of the actual state of the nanomaterial during operation, mechanistic models risk being based on incorrect assumptions. Table 1 provides an overview of selected *in situ* and *operando* characterization techniques and their primary sensitivity to different classes of dynamic transformations in electrochemical nanomaterials, highlighting the complementary nature of these methods in capturing phase, surface, morphological, and interfacial changes under operating conditions. However, researchers should exercise caution when using these complementary methods, as some samples are sensitive to electron, X-ray, or laser beams. Therefore, selecting appropriate measurement parameters is essential to avoid beam-induced damage and, consequently, incorrect conclusions.^88–90^

**Table 1:** Correlation between *In Situ*/*Operando* Characterization Techniques and Dynamic Transformations in Nanomaterials during Electrochemical Operation

Technique	Dynamic changes it probes
*Operando* XAS (XANES/EXAFS)	Oxidation state evolution; local coordination changes; electronic structure rearrangement
*Operando* XRD	Phase transitions; bulk crystallographic reconstruction; long-range structural evolution
Electrochemical Raman spectroscopy (including SERS)	Surface reconstruction; intermediates formation; adsorbate evolution
EC-TEM	Morphological evolution; atomic-scale structural dynamics (atom migration, aggregation, dispersion); particle growth
SECM	Local reactivity changes; spatial heterogeneity of activity; surface deactivation/reactivation dynamics
ICP-MS (online/flow analysis)	Dissolution and redeposition of active species; elemental leaching into electrolyte
Dark-field/optical microscopy	Macroscopic morphological changes; bubble formation; detachment processes
Near ambient pressure XPS	Surface composition and oxidation state changes; adsorbate coverage (quasi-operando)

For electrochemists using nanomaterials, it is essential to understand that they should be viewed as dynamic systems, continuously evolving in response to the applied potential and electrolyte environment. Recognizing and quantifying these changes, before, during, and after operation, is essential for establishing reliable structure–property relationships. Without this perspective, electrochemical measurements risk being interpreted in terms of static models that do not reflect the true, time-dependent nature of the nanomaterial under operating conditions.

### 2.5 The Solid–Liquid Interface Is a Dynamic Interphase, Not a Sharp Boundary

In electrochemical studies of nanomaterials, the solid–liquid interface is often treated as a sharp boundary between an electrode and an electrolyte. This simplification is useful conceptually, but it can be misleading for nanostructured and dynamically evolving electrodes. In such systems, the reactive zone may extend across a finite interphase that includes the outermost atomic layers of the solid, reconstructed or transformed surface/subsurface regions, adsorbed intermediates, passivating or catalytically active surface layers, specifically adsorbed ions, oriented interfacial water, local electric fields, and, in gas-involving reactions, local gas domains or nanobubbles.^91–96^

This means that the electrode material and the electrical double layer are strongly coupled. The electronic structure, work function, crystallographic orientation, defect density, oxidation state, hydrophilicity, and adsorbate coverage of an electrode can influence interfacial water orientation, ion distribution, local acidity/alkalinity, electric fields, and proton or hydroxide transfer pathways.^91–94,97,98^ Conversely, electrolyte ions and interfacial molecules can modify surface charge, adsorbate binding, reconstruction, apparent transfer coefficients, and even the electronic structure near the surface.^92–95,97–99^ Therefore, apparent activity and selectivity should be understood as properties of the coupled electrode–electrolyte interphase, rather than of the solid catalyst or electrolyte alone.

Recent studies illustrate this point clearly. Cation-dependent changes in interfacial water structure on Pt have been shown to influence alkaline hydrogen electrocatalysis, while cation-limited hydroxide transport at transition-metal-decorated Pt surfaces has been proposed to contribute to asymmetric HER/HOR kinetics.^97,98^ Likewise, atomistic simulations of electrified Pt(111)–water interfaces indicate that the electronic response of the metal is not necessarily confined strictly to the solid surface, but may extend into the first interfacial water layers.^95^ These examples show that catalyst descriptors based only on the electronic or binding properties of the dry solid are incomplete if they neglect the double layer, interfacial water, and ion distributions. Similarly, electrolyte descriptors based only on bulk pH, nominal cation identity, or ionic strength are incomplete if they ignore how the electrode surface modifies the local interfacial environment. This finite-interphase concept is also essential for dynamically reconstructing electrocatalysts.^100^ For example, in many transition-metal-based OER catalysts, where the active phase is a potential-induced surface or near-surface oxide/oxyhydroxide layer rather than the as-synthesized material, the reactive zone may include both the reconstructed surface and the subsurface region that supplies ions, defects, charge carriers, or lattice oxygen.^46,101–104^

Gas-involving electrocatalytic reactions further broaden the definition of the reactive zone. During CO_2_ reduction, hydrogen evolution, nitrogen reduction, oxygen evolution, and related processes, gaseous reactants or products may accumulate near the surface, inside hydrophobic pores, at defects, or within rough nanostructured films. Local gas domains and nanobubbles can modify mass transport, block or expose active sites, change local supersaturation, and alter the composition of the interfacial region.^105,106^ In CO_2_ electroreduction, for example, nanobubble-infused electrolytes have been reported to enhance mass transfer, illustrating that gas domains can directly influence measured performance.^106^ In addition, radical formation associated with bulk nanobubbles or microbubble-treated water has been reported, suggesting that bubble-related phenomena may sometimes affect surface chemistry beyond simple mass transport.^107^ Although such effects will depend strongly on the reaction, electrolyte, potential, and cell geometry, they reinforce the need to avoid treating the reactive interface as a simple line.

The practical implication is that mechanistic claims should define which part of the interphase is being probed. When possible, electrochemical data should be complemented by *operando* or *in situ* spectroscopy, microscopy, local pH or ion-concentration measurements, differential electrochemical mass spectrometry, scanning probe methods, or other techniques capable of resolving changes in surface composition, interfacial water, adsorbates, ions, gas domains, and local reaction environments.

## 3 Advice to Materials Scientists from Electrochemists

### 3.1 Need for Fundamental Understanding!

The activation of atoms and molecules by light, both to bring about changed or enhanced reactivity and, in the form of spectroscopy, to characterize the identity and electronic properties of the species under irradiation, is a highly familiar and widely undertaken activity by materials scientists. It is important, however, to recognize that electrochemistry is both qualitatively and quantitatively different from photochemistry. Specifically, the latter involves absorption of light and transitions between specific energy levels of the species investigated. As such, the frequency of the light absorbed must match the energy separation between the start and end energy levels. Light of too low, or too high a frequency, does not bring about the sought transition. Further, when the light is absorbed, the species is unchanged as a chemical entity. However, in the case of electrochemistry, the application of a suitably oxidizing or reducing electrical potential to the target entity *via* an electrode leads to the removal (oxidation) or the addition (reduction) of an electron from the electrode. Thus, the initial product is either a cation or an anion (if the starting species is neutral), which is a chemically distinct (in terms of number of electrons) species. Moreover, and importantly, provided the voltage of the electrode exceeds a certain threshold value, all voltages in excess of the threshold realize the formation of the cation or anion.

This consideration highlights an important contrast between photochemistry and electrochemistry. In photochemistry, tuning the frequency (energy) of the light to an appropriate energy gap in the target selectively activates the species, while light of lower or higher energy is ineffective. In electrochemistry, however, once the threshold potential for electron transfer into or out of the molecule is exceeded, the anion or cation is produced at all potentials applied (Figure 4). Thus, the potential used in electrochemistry should be seen as indicating a threshold above which activity takes place, in contrast to photochemistry, where the choice of energy (frequency) can selectively activate, or not, the target. To be explicit, for example, a chlorophyll molecule can be selectively activated (and fluorescence induced) by excitation at 420 nm, but excitation at much higher or lower frequencies is ineffective. In contrast, in electrochemistry, if the application of a certain potential to a molecule brings about its oxidation or reduction, then that reaction will continue to proceed at all larger (more positive for an oxidation, more negative for a reduction) potentials. There is no switching off at higher energies as seen in the photochemical case, but with the proviso that further oxidation (for example, to a dication) or further reduction (to a dianion) might take place *via* reaction of the first formed cation or anion. A key distinction between photochemistry/spectroscopy and electrochemistry is that, in the former, activation can take place by the careful selection of the light energy (frequency), and this allows some molecules to be selectively activated in the presence of other which absorb light at different (both higher and lower) frequencies. In contrast, because of the threshold nature of the redox activity, in electrochemistry, if a given potential is applied, then this will oxidize or reduce all species present which are above the threshold. Thus, the electrochemical “activation” is less selective than the photochemical process.

**Figure 4:**
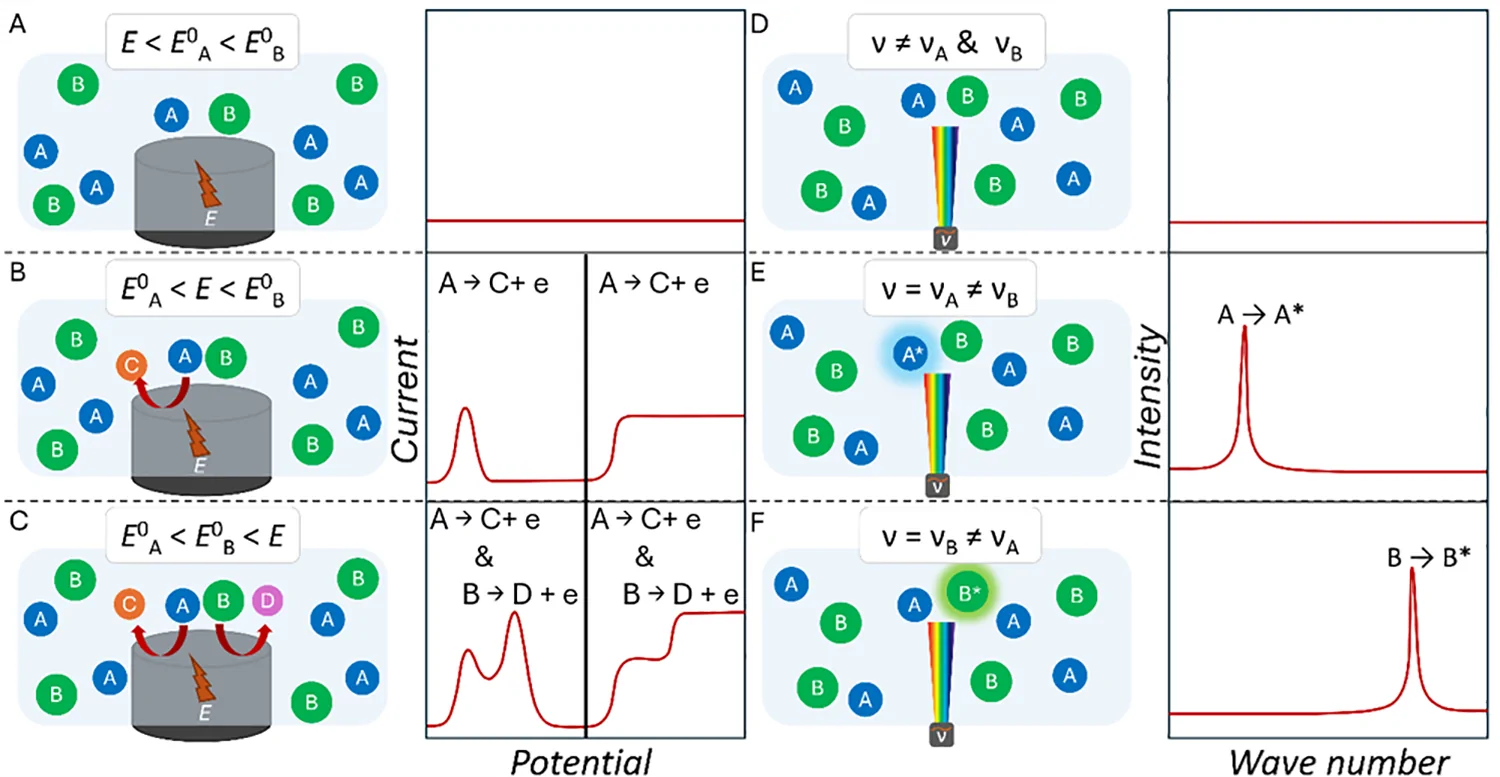
Schematic comparison of electrochemical and photochemical processes. (Left) In electrochemistry, reactivity is governed by the applied potential (E); (A) no transformation occurs below the redox threshold, while (B and C) species are activated once their standard potentials (E_A_^0^, E_B_^0^) are exceeded. Both transient (peak-shaped) and steady-state (sigmoidal) voltammetric responses are possible, depending on the technique; however, in all cases, electron transfer continues at potentials beyond the threshold rather than switching off. (Right) In photochemistry, reactivity is dictated by excitation energy; (D) excitation does not occur outside the appropriate energy range and (E and F) only species with absorption matching the incident frequency (ν) are selectively excited, producing band-shaped responses; ν_A_ and ν_B_ denote the characteristic wavenumbers at which species A and B absorb or interact with the radiation, respectively.

A further subtlety emerges in terms of the meaning to be assigned to the values given for the photochemical or electrochemical activation. In the former case, the wavelength of the light used will probably correspond to an absorption band of the target species, usually the wavelength of maximum absorption as revealed by an absorption spectrum, a plot of absorption *vs* wavelength, which shows different absorption bands. In the electrochemical case, the potential used for transformation may exceed the minimum required, and indeed, the greater the potential, the greater the Faradaic current (corresponding to transfer of electrons at the electrode/solution interface) up to a point at which the process becomes sufficiently fast that the rate-determining step becomes the rate of transport *via* diffusion and convection of the target species to the electrode. In this case, it is informative to examine a “voltammogram”, a plot of current *vs* the potential applied to an electrode, generally obtained by scanning the potential from a defined start value in either a cathodic or anodic direction at a fixed scan rate (V s^–1^). Typically, this will be either peak or sigmoidal shaped. The potentials beyond the peak or corresponding to the plateau of the sigmoid are associated with transport-controlled conditions. A plateau appears when steady-state mass transport is established, either by convection, as in a rotating electrode or by radial diffusion at an electrode of microdimensions in size. Note that, in contrast to spectroscopy, the start potential used for recording the voltammogram must generally be carefully selected so as to correspond to zero Faradaic current, so as to avoid artifacts, including the depletion of reactants if a large potential is applied immediately.

### 3.2 Electrocatalysis or Not?!

As mentioned in Section 2.3, particles of the electrocatalyst are assembled into a (often drop-cast) film or composite material to make an electrode, typically a macroelectrode, and the electrode response is highly sensitive to the loading in an emphatically nonlinear manner. At very high coverages, multilayers of particles form on the electrode surface, having some degree of porosity. Accordingly, electron transfer can take place both at the surface of the porous layer and within it, introducing coupled kinetic and mass-transport effects across the film thickness. Porosity adds a critical additional level of complexity, as the electrolyte occupies a network of internal pores. Mass transport then occurs on (at least) two length scales: diffusion from the bulk electrolyte to the outer film surface, and diffusion within the internal pore volume of the film. This leads to a complex interplay of transport to and inside the layer, combined with the electrode kinetics of the particles and, if some reactant survives diffusional passage through the layer, the kinetics of reaction at the underlying substrate (electrode). The complexities have been modeled by Yang et al.^108^

Modeling of regular nanoparticle packings shows that, for realistic pore sizes and scan rates, the concentration inside the pores can reach Nernstian equilibrium on time scales of hundreds of microseconds, much faster than typical voltammetric scans on flat electrodes.^109^ In this limit, the internal electrolyte behaves as a thin layer with rapid internal equilibration but limited exchange with the bulk solution. This leads to two distinct mass-transport regimes: (i) thin-layer-like behavior at high scan rates and/or thick porous layers, and (ii) semi-infinite diffusion behavior for thin films or slow scan rates (Figure 5).

**Figure 5:**
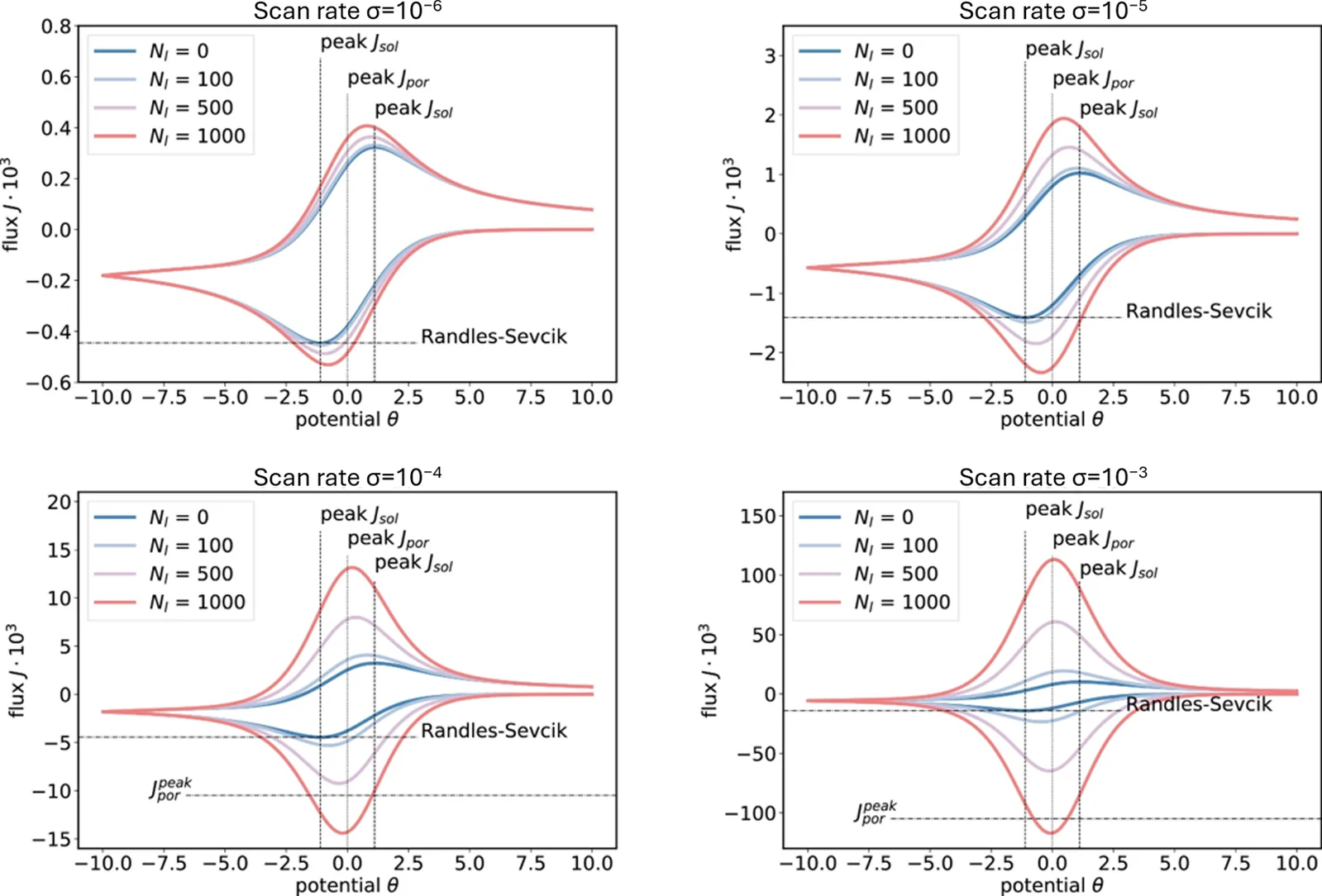
Dimensionless voltammograms calculated for the total electrode current (*J*), evaluated for four different scan rates (σ) and various numbers of deposited layers (N_l_). The depicted scan rates correspond to dimensional scan rates of 2.57 mV s^–1^, 25.7 mV s^–1^, 257 mV s^–1^, and 2.57 V s^–1^, if evaluated for a diffusion coefficient *D* of 10^–9^ m^2^ s^–1^ and a sphere radius *r*_s_ of 100 nm. Adapted from ref 109. Copyright 2020 Elsevier.

One challenge for the experimentalist is that the voltammetric responses from the target reactant species contained inside the porous layer undergoing diffusion to the catalyst surface under so-called thin-layer conditions resemble that seen from an adsorbed species, corresponding to a quite different electrode reaction mechanism.^110^ In both cases, the voltammetric wave is shifted to lower potentials than seen for reaction on a planar surface under semi-infinite diffusion conditions for the same electrochemical rate constant. It is therefore easy to (mis)­interpret the voltammetric response conferred by virtue of electrolysis taking place within a porous environment as an electrocatalytic effect (rather than a mass transport effect) due to the chemical nature of the particles, being reflected in a change of the electrochemical rate constant. In such cases, comparing cyclic voltammograms at different scan rates and of films with systematically varied thickness and porosity is straightforward and informative. Thin-layer control tends to give peak or charge responses proportional to scan rate (υ), whereas semi-infinite diffusion gives the υ^1/2^ scaling.

More generally, the rate of an electrocatalytic reaction using an electrode modified with an ensemble of particles reflects both the surface area of the catalyst and the pertinent electrochemical rate constant, but also the porosity, thickness, and resistivity of the catalyst layer. Figure 6 shows finite-element simulations of a model electrode comprising several layers of catalytic nanoparticles embedded in a binder, mapping out regimes where

- All layers participate and the film behaves almost ideally
- Inner layers become mass-transport limited and effectively switch off at higher currents
- Outer layers are suppressed by ohmic drops, so only material close to the current collector is active, or
- Realistic combinations of resistance and hindered diffusion yield responses in which different parts of the film operate under different control (kinetic, mass-transport, or ohmic) at the same applied potential.^111^

**Figure 6:**
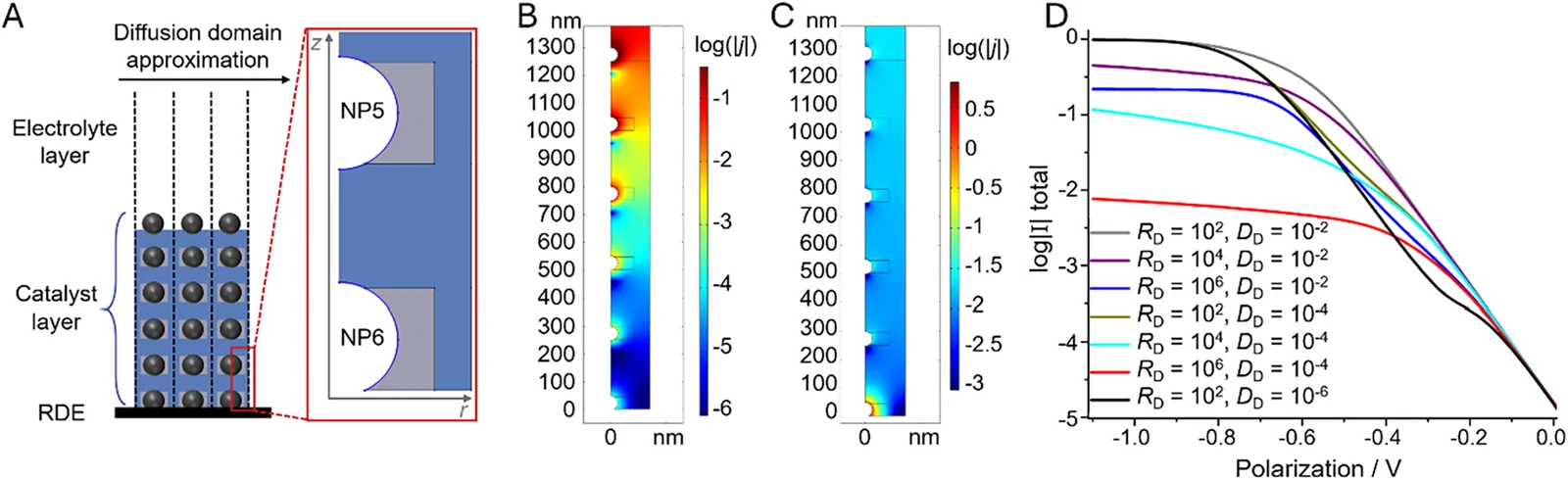
(A) The schematic cross-section of a nanoparticle film immobilized on an RDE and the used model geometry for numerical simulations. The simulated logarithm of the total flux magnitude at 25 nm Au nanoparticles (scan rate = 5 mV s^–1^), NPs distancing = 250 nm, *k* ^0^ = 6.4·10^–7^ cm s^–1^ and α = 0.46 (B) at an electrode polarization of −1 V, and (C) at an electrode polarization of −0.72 V and dimensionless resistance scaling factor of 10^4^. (D) Comparison of logarithmic dimensionless currents *versus* the electrode polarization of linear sweep voltammetry (LSV) curves with various combinations of dimensionless resistance (*R*_D_) and dimensionless diffusion coefficient (*D*_D_). Adapted from ref 111. Copyright 2020 Elsevier.

These regimes are reached by varying the effective resistance parameter, which describes how resistive the film is relative to an ideal conductor and the effective diffusion parameter, which describes how slow the diffusion is in the film electrolyte relative to the bulk solution.

These simulations show that different regions of the film can simultaneously operate under kinetic, mass-transport, or ohmic control, depending on position and operating conditions. Crucially, in many of these regimes, the overall current–potential curve still exhibits a visually convincing Tafel region, *i.e.*, a straight line over one or two decades of current on a semilog plot. However, the corresponding slope and intercept are strong functions of film thickness, porosity and resistivity, not pure kinetic constants of a single, well-defined interface. This is consistent with experiments on poorly conductive catalysts such as MoS_2_, where spatially heterogeneous activity emerges depending on carbon content and film thickness.^112^

Interestingly, the IUPAC Gold Book defines a catalyst as a “substance which increases the overall rate of reaction without modifying the overall standard Gibbs Energy change in the reaction”. This definition in terms of rate, rather than rate constant, suggests that not only increasing the rate constant but also the area (or amount of catalyst) serves to catalyze a reaction. In reality, chemists are interested in lowering the activation energy for the sought electrochemical process and thereby increasing the (heterogeneous) rate constant.

This perspective complements the earlier emphasis on impurities and dominant reactive centers. In one case, a chemically distinct minority phase (*e.g.*, a residual Fe oxide) can dominate the response of a nominally inert carbon matrix. In the other, specific regions of a porous film (*e.g.*, layers near the current collector, or pores with particularly favorable transport pathways) can dominate the macroscopic current. In both situations, naive interpretations of activity per surface area or per mass become questionable if the underlying structure is not understood.

Taken together, these examples convey a simple but important message: porous composite electrodes cannot be treated as black boxes that magically deliver a unique, geometry-independent activity descriptor. Instead, any electrochemical observable (current density, Tafel slope, TOF, onset potential) is jointly determined by intrinsic kinetics and electrode architecture. Accordingly, systematic variation of thickness, porosity, coverage and composition is needed to identify which part of the response is robust and which is architecture dependent. By scanning coverage over several orders of magnitude, a coverage window could be identified, in which the catalytic current is genuinely proportional to the catalyst surface. Only in that window, it is meaningful to normalize current by electrocatalytic surface area and directly interpret the result as an intrinsic rate constant.^113,114^

No universal “ideal” catalyst loading can be prescribed, because it depends on particle size, interparticle distance, porosity, electronic conductivity, electrolyte transport properties, hydrodynamics, and experimental time scale. Practically, the ideal loading range for reporting intrinsic activity is therefore the system-specific linear-coverage regime: the loading must be low or thin enough to avoid severe concentration gradients inside pores, and ohmic drops across the film, but high enough to provide a reproducible signal above the noise level. This regime should be established experimentally rather than assumed, for example, by measuring a loading series and identifying the window in which the catalytic current scales linearly with catalyst coverage or independently determined ECSA, while the ECSA-normalized activity, apparent Tafel slope, and selectivity remain insensitive to changes in loading. Data recorded outside the linear-coverage window remain valuable for assessing practical electrode performance, but they should be reported as architecture-dependent electrode performance rather than as intrinsic catalytic activity. Thus, if the apparent Tafel slope or overpotential changes significantly when the film thickness, ionomer fraction, porosity, or coverage are varied within a realistic range, this variation should be reported explicitly, as it reveals possible ambiguity of this metric for the specific system and testing parameters. Complementary single-entity methods, particularly nanoimpact electrochemistry, can provide an additional route to probe catalytic activity at the individual-particle level and thereby help distinguish particle-specific intrinsic behavior from ensemble-averaged, film-architecture-dependent responses.^115,116^

Once we acknowledge that porous films behave as inhomogeneous, coupled kinetic–transport–ohmic systems, it is inconsistent to treat the resulting Tafel parameters or rate constants as if they were geometry-independent constants with negligible uncertainty. This naturally motivates the need for rigorous error analysis and transparent reporting of architecture-dependent uncertainties. If the apparent Tafel slope, overpotential or rate constant depends sensitively on coverage, film thickness or porosity, this variability must be reflected in the way data are reported. In interpreting voltammetric data so as to identify the causes of any possible catalysis, it is essential to deconvolute contributions from changing surface areas, electrochemical rate constant and film/composite porosity. A protocol for this has been defined and applied to the Pd-nanoparticle-catalyzed electro-oxidation of hydrazine, with both surface area and kinetic contributions to the catalysis quantified.^117^

### 3.3 Particle and Material Porosity

As mentioned above, electrochemical responses reflect both the rate of heterogeneous electron transfer (“electrode kinetics”) and the mass transport to the catalytic particle(s). In the case of individual, isolated, single particles, the latter reflects both size and shape as well as the particle porosity. All three parameters are important when characterizing materials under study, especially if, for example, ML approaches are to make use of reported data so as to successfully predict optimal structures as synthetic targets. Size influences electrochemical measurements since, in general, the smaller the electroactive center, the greater the diffusive flux and hence the rate of reactants to the particle and of products away, as reflected in the commonly well-appreciated contrasts between micro- and macro-electrodes. However, size is not the only parameter needing characterization. The rates of diffusion to isolated spheres, cubes, cylinders, *etc.*, and similar-shaped particles located on a plane (for example, an electrode) show geometry-dependent responses;^118,119^ hence it is vital to characterize particle shape. Moreover, the entities themselves can be heterogeneous. For example, investigation of isolated partially active nanocubes with different numbers of electro-active faces showed different diffusional responses according to how many faces were active and to the relative location of the active faces.^120^ Importantly, the current was not linearly proportional to the active surface area since the faces are not diffusionally independent of one another, as the flux to one face reflects the activity or otherwise of nearby faces. In general, the shape of particles can control the degree of electrochemical reversibility with concave surfaces, as opposed to convex, promoting the apparent reversibility.^121^

Single particle porosity can be experimentally challenging to measure, but it is very important since electrochemical reactions can and do take place on internal surfaces of the particles as well as on their external surfaces. For example, the underpotential deposition of hydrogen on the internal surfaces of (individual) Pt nanoparticles has been demonstrated and used for measuring the surface area (external + internal) of the particles. Such measurements are made using the nanoimpact technique in which individual nanoparticles, as part of a suspension in solution, diffusionally encounter a microelectrode with a selected imposed potential.^122–124^ This approach offers major benefits for characterizing porosity at the single particle level.^125,126^ This contrasts with the widely used approach of using gas adsorption and the BET isotherm to infer surface areas of particles. In this context, it is important to recognize that only certain types of adsorption isotherms can be analyzed for porosity; it is not a measurement to be treated as routine, rather, if it is used, then the full adsorption isotherm needs to be published and, following the internationally recommended approach,^127^ the isotherm needs to display the characteristics of Type 2 or Type 4 isotherms. Note that some software associated with automated BET analysis may report numbers for BET-based surface areas regardless of the adsorption type and whether the reported isotherm contains the relevant information. Thus, in addition to the isotherm, we suggest that the specific model of porosity used (for example in respect of pore shape) is reported. An alternative, simple approach involves the adsorption of molecules such as dopamine or methylene blue from the solution phase^128^ or measurement of the electrical double-layer capacitance of the respective porous particle.^129^

### 3.4 Practical Limits of Electrochemical Equations

Electrochemical equations provide an essential framework for interpreting experimental data, offering relationships between measurable quantities and insights into the underlying physicochemical processes. However, their applicability relies on a set of simplifying assumptions that are not always fulfilled when using actual, nanoscale materials.

A typical example is the Levich equation, which describes mass transport under controlled hydrodynamic conditions at a rotating disk electrode (RDE) and allows studying the kinetics of electrochemical reactions.^130–132^ The RDE geometry imposes a well-defined convective flow toward the electrode surface and a controlled diffusion layer. Under these conditions, the diffusion-limited current *I*_lim_ [A] is given by

1 $$I_{\lim}=\mathrm{kzFA}D^{2/3}\omega^{1/2}\upsilon^{-1/6}c_{0}$$

where *k* is a numerical factor, *z* is the electron number of an electrochemical reaction, *F* is the Faraday constant [C mol^–1^], *A* is the electrode surface area [m^2^], *D* is the diffusion coefficient of the electroactive substance [m^2^ s^–1^], ω is the angular velocity of the electrode [rad s^–1^], ν is the kinematic viscosity of the solution [m^2^ s^–1^], *c*_0_ is the concentration of the electroactive substance in the bulk solution [mol m^–3^].^131,133^ This expression is derived assuming laminar flow and a simplified description of the velocity profile normal to the electrode surface, valid when the diffusion layer is thin relative to the hydrodynamic boundary layer (*i.e.*, at high Schmidt numbers). A key assumption is that the electrode is uniformly accessible, so that mass transport can be treated as one-dimensional and normal to the surface.^134^ Further, any possible finite homogeneous chemical kinetics are neglected.

In practice, these assumptions are only approximately satisfied. The condition of uniform accessibility is strictly valid only for an electrode of infinite radius.^135^ The finiteness of real electrodes introduces edge effects and radial diffusion contributions that become increasingly relevant at low rotation rates.^136^ As a result, the current can deviate from the ideal Levich behavior, typically exceeding the predicted values due to enhanced mass transport at the electrode edge.^134^ Moreover, nanostructured materials may include porous architectures, nanoparticle aggregates, and heterogeneous catalysts that introduce internal, intricate transport pathways and spatial variations of the reactant concentration, so that the mass transport is no longer governed solely by an external hydrodynamic diffusion layer. Conversely, the transport of reactants and reaction products occurs across multiple length scales and involves multiple processes, such as bulk diffusion and pore diffusion. As a consequence, the measured current reflects the convolution of these contributions, rather than a single, well-defined transport regime. Indeed, significant deviations from Levich predictions can be observed for porous rotating disk electrodes.^137^ In practical catalyst layers, exemplified by the carbon-supported platinum for proton exchange membrane fuel cells, additional factors, such as binder effects and oxygen diffusion within the film, further complicate the extraction of intrinsic kinetic parameters from RDE measurements.^138^

To account for both kinetic and transport contributions, the Koutecký–Levich equation, originally introduced by Alexander Frumkin,^139^ is commonly employed

2 $$\frac{1}{I}=\frac{1}{I_{k}}+\frac{1}{I_{\lim}}$$

where *I* is the measured current [A] and *I*_*k*_ is the kinetic current [A]. Although this framework is used to separate kinetic and mass transport effects, its validity depends on the underlying assumptions of uniform activity and well-defined transport pathways.^140^ Notably, when applying Koutecký–Levich analysis to electrodes modified with micro- or nanoparticles, the inferred rate constants reflect an apparent rate constant *k*_app_, which depends on the ratio ψ of total electroactive surface area to the geometric electrode area. For ψ > 1, *k*_app_ may overestimate the true rate constant *k*_0_, potentially leading to a false inference of catalytic enhancement, whereas for ψ < 1, *k*_app_ underestimates *k*_0_, possibly masking genuine catalytic effects.^141^ Furthermore, the Koutecký–Levich formalism strictly applies to simple, single-step electron-transfer mechanisms, and its use for multistep reactions, such as the oxygen reduction reaction, may lead to incorrect estimates of kinetic parameters and electron transfer numbers.^142,143^

Another relevant equation in electrochemistry is the Tafel equation, a relationship between the potential *E* and the current density *j*

3 E=a+b×log|j|

where *b* represents the Tafel slope.^144^ This equation suggests an exponential relationship between *E* and *j*, later rationalized by the Butler–Volmer equation.^145,146^ Importantly, the Tafel slope provides insights into charge-transfer kinetics and, in some cases, the rate-determining step of a reaction, unravelling the reaction mechanism. Nevertheless, its interpretation is often more complex than commonly assumed. Indeed, it relies on a number of stringent assumptions, including negligible mass-transport limitations (typically addressed by using an RDE), the absence of backward reactions, complete compensation of ohmic losses, and a constant number of active sites that does not vary with the applied potential. In addition, it is typically assumed that capacitive currents and variations in surface coverage of reaction intermediates do not significantly contribute to the measured response.^147^

In practice, these conditions are rarely fulfilled in nanomaterials. Apparent Tafel slopes are frequently affected by nonkinetic contributions, including mass transport limitations, local concentration gradients, bubble formation, and potential drops across porous or resistive catalyst layers. These effects are particularly relevant in nanostructured and high-surface-area electrodes, where internal diffusion pathways and heterogeneous current distribution complicate the interpretation of current–potential relationships. For example, in gas-evolving reactions such as HER, locally generated gas can accumulate within the catalyst layer, modifying the effective reaction environment and distorting the measured current. This can result in artificially low Tafel slopes at low overpotentials, due to partial compensation by reverse reactions, or artificially high slopes at higher overpotentials, where gas accumulation suppresses further current increase.^148^ Similarly, variations in catalyst loading, film thickness, or porosity can influence the apparent slope.^116^

These challenges are compounded by the methodology used to extract Tafel slopes. Values derived from linear sweep voltammetry may differ from those obtained by chronoamperometry or from frequency-domain techniques such as EIS, particularly when steady state is not achieved. Moreover, the common practice of selecting an arbitrary potential window for linear fitting can introduce significant bias if the *E vs log|j|* relationship is not strictly linear. In particular, both experimental observations and microkinetic models show that the slope can vary with the potential due to changes in intermediate surface coverage, double-layer effects, or shifts in the rate-determining step. In many processes, such as alkaline HER or CO_2_ reduction reaction, no clear region exists in which the Tafel slope remains constant, making the mechanistic interpretation ambiguous. In contrast, well-defined systems, such as HER on platinum RDE in acidic media, can exhibit a stable slope over a limited current range.^149^ More robust approaches, such as differential Tafel analysis^150^ or plotting the local (instantaneous) Tafel slope as a function of potential or current, provide a more reliable way to identify regions where the slope is constant and thus kinetically meaningful.

A general description of electrode kinetics is commonly provided by the Butler–Volmer formalism, which relates the current flux *j* to the applied potential *E*. In its classical formulation, this framework has historically been used to extract anodic α_*a*_ and/or cathodic α_*c*_ transfer coefficients from Tafel analysis.^151^ Unfortunately, using expressions containing the product of the transfer coefficient and the total number of electrons transferred (α·*z*) has led to misleading conclusions. Indeed, many studies mistakenly calculated *z* from fitted *α·z* by assigning a specific value to α (typically 0.5). This approach is problematic partly because the Butler–Volmer formalism is usually derived only for a single elementary electron-transfer step. In contrast, most electrocatalytic reactions of interest involve many steps, often involving multiple electron transfers, and each with its own formal potential and hence “overpotential”. Therefore, this practice has been strongly discouraged by IUPAC.^151^ To avoid this issue, for the reaction *A* + *z*e^–^ ⇄ *B*, the Butler–Volmer equation can be expressed as the difference of two terms for the total flux, *j* [A m^–2^], one each for the anodic (*j*_*a*_) and cathodic (*j*_*c*_) processes

4 $$j=j_{a}-j_{c}$$

with

5 $$j_{a}=k_{a}F\exp\left(\left(z^{\prime }+\alpha_{a}\right)FE/RT\right)c_{B}$$

and

6 $$j_{c}=k_{c}F(\exp\left(-\left(z^{\prime \prime }+\alpha_{c}\right)\mathrm{FE}/\mathrm{RT}\right)c_{A}$$

where *k*_*a*_ and *k*_*c*_ are heterogeneous rate constants [m s^–1^], *z*′ is the number of electrons transferred before the rate-determining electron-transfer step in the anodic direction with *z*″ the corresponding quantity in the cathodic direction, α_*a*_ and α_*c*_ are the anodic and cathodic transfer coefficients usually with values between zero and unity, *c*_*A*_ and *c*_*B*_ are the concentrations of A and B [mol m^–3^].^152^ Note that *z*′ and *z*″ do not necessarily sum to *z* since the oxidations and reductions typically occur at quite different potentials (for irreversible processes) and the mechanism can likely change with potential.

It is important to reflect that in many multistep electrocatalytic reactions, such as oxygen and CO_2_ reduction, the value of *z* (or the appropriate formal potential) is, in the absence of coulometry, unknown, notably due to the presence of multiple possible pathways and products. Accordingly, eq ([Disp-formula eq4]) is defined to be independent of *z*. Moreover, the IUPAC assessment by Guidelli et al. proposes a better definition of the anodic and cathodic transfer coefficients as

7 $$z^{\prime \prime }+\alpha_{c}=-\frac{\mathrm{RT}}{F}\frac{d\ln\left|j_{c}\right|}{dE},\;z^{\prime }+\alpha_{a}=\frac{\mathrm{RT}}{F}\frac{d\ln j_{a}}{dE}$$

Using eq ([Disp-formula eq7]), the estimation of *z*″ *+* α_*c*_ and *z*′ + α_*a*_ is independent of *z*.^151^ Further note that in some reactions, for example CO_2_ reduction, there are parallel pathways leading to diverse products so that a single equation of the form of eqs ([Disp-formula eq4]–6) is unlikely to be physically applicable.

When studying nanomaterials, it is crucial to recognize that electrochemical equations like Levich or Tafel are ideal frameworks. Real electrodes, particularly porous, nanostructured, or heterogeneous systems, often violate their underlying assumptions, meaning that measured currents and slopes may reflect combined effects of transport, surface coverage, and local reaction environments. It is important to recognize that Tafel slopes are structure-sensitive and depend on the physicochemical state of the electrode surface. In this regard, the use of well-defined model systems, such as single-crystal electrodes under controlled hydrodynamic conditions, provides a valuable benchmark for discriminating intrinsic kinetics from extrinsic effects. More advanced analyses, including microkinetic modeling, temperature-dependent studies, and consideration of interfacial phenomena such as specific adsorption or double-layer structure (*e.g.*, Frumkin corrections), are often required to extract meaningful mechanistic insight.^147^ Careful consideration of electrode structure, mass transport, and measurement protocols must be embraced to ensure that observed kinetics correspond to intrinsic catalytic behavior rather than artifacts of the nanomaterial structure or the experimental setup.

### 3.5 Need for Error Analysis and the Value of Negative Results

The discussion above shows that electrocatalytic performance extracted from porous, composite films is a convoluted quantity, reflecting not only intrinsic kinetics but also contributions from porosity, thin-layer effects, and resistive losses. It follows that electrochemical data from such systems cannot be meaningfully compared or fed into models without an explicit treatment of uncertainty, and error bars and negative results are central to such treatment. This is particularly important in the context of AI-ready data sets. If the underlying measurements are poorly constrained and their uncertainties are invisible, then even the most sophisticated algorithms will simply learn artifacts of film architecture, impurities and cell design, rather than genuine structure–activity relationships.

This is whereas electrochemical data in the materials literature are still too often presented as if they were exact numbers; single Tafel slopes, single overpotentials at 10 mA cm^–2^, single turnover frequencies, oftentimes quoted without (an appropriate) indication of uncertainty. For complex, multicomponent catalyst films, this gives a very misleading impression of precision.

In reality, there are at least three distinct sources of variability (Figure 7) which should be reflected in the way data are reported:

- Electrochemical measurement uncertainty; instrumental noise and drift (potentiostat calibration, reference electrode stability), uncompensated resistance, gas bubbles, temperature variation, capacitive currents, *etc*.
- Materials and electrode variability; batch-to-batch differences in composition, phase, defect chemistry, particle size distribution, porosity and ink formulation, and differences in film thickness, ionomer and carbon content, *etc*.
- Model and data-treatment uncertainty; how kinetic parameters (Tafel slopes, exchange current densities), ECSA and transport corrections are extracted when the underlying assumptions (homogeneous planar electrode, semi-infinite diffusion, negligible film resistance) are violated.

**Figure 7:**
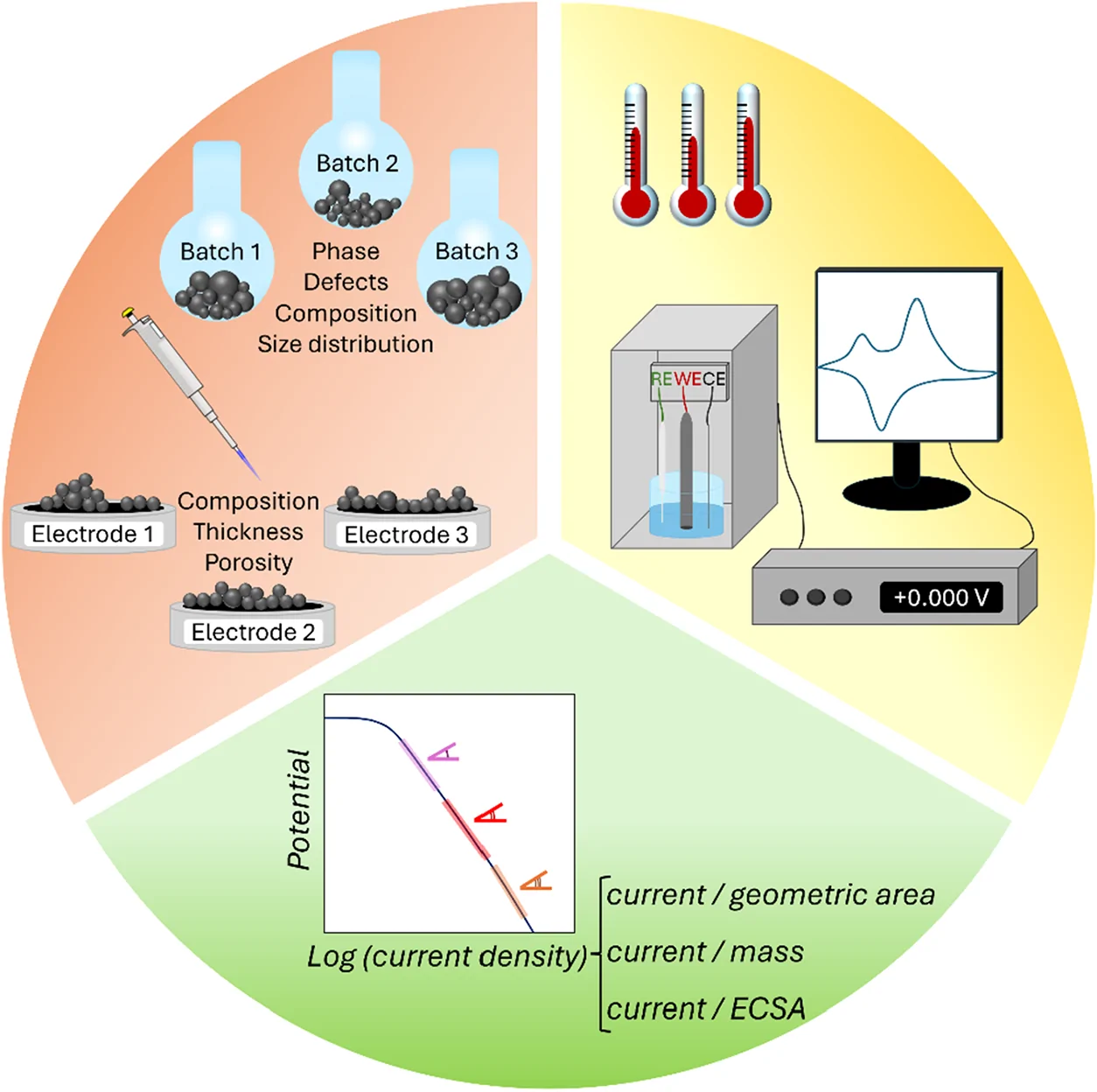
Schematic representation of sources of variability and uncertainty in catalyst evaluation.

If materials scientists wish to publish electrochemical performance metrics that are useful beyond a single laboratory, these three levels of uncertainty should be quantified and communicated. For typical benchmarking quantities such as the potential at a defined current density, Tafel slopes, turnover frequencies, or double-layer capacitances, the relevant variation is not the noise of a single voltammogram, but the spread obtained when the material is independently synthesized several times, electrodes are freshly prepared from each batch (separate inks, separate depositions, separate membrane–electrode assemblies), and the same electrochemical protocol is applied using independently calibrated reference electrodes and electrolyte batches.

Only then do the error bars include the variability that would be encountered when another laboratory attempts to reproduce the synthesis and the measurement. In other words, “*n*” should, as far as reasonably possible, count independent syntheses and electrode preparations, not just repeated scans of the same film. The latter mostly probes short-term drift and catalyst aging, which is useful but conceptually different and should be labeled as such.

Reporting a single Tafel slope to two decimal places, without considering the variability introduced by film architecture, is inappropriate and potentially misleading. A more transparent approach is to report a range, for example, 55–60 mV dec^–1^ across well-characterized films and to specify whether this spread arises primarily from structural variation, data analysis, or both. As discussed in Section 3.4, when the *E vs* log*|j|* relationship is not strictly linear, the apparent Tafel slope should preferably be evaluated as a local or differential quantity, for example by determining the gradient of the log*|j|*–potential curve at specified potentials and reporting the corresponding local Tafel slope (d*E*/dlog*|j|*) as a function of potential. Equivalently, an apparent transfer coefficient may be reported as a function of potential.

In parallel, the reproducibility of the reported values should be assessed using independently prepared electrodes, ideally from different ink preparations or material batches, analyzed using the same predefined protocol. This reproducibility range captures sample-to-sample, film-to-film, and measurement variability and may be reported as a mean value with standard deviation or confidence interval. The potential dependence of the local Tafel slope and the statistical scatter between replicate electrodes should be reported separately rather than collapsed into a single unexplained number. If the local slope varies substantially over the relevant potential range, the mechanistic interpretation of a single Tafel slope should be avoided or clearly qualified. A more rigorous reporting strategy is therefore to provide either the local Tafel slope as a function of potential or, where a sufficiently constant region exists, a representative slope range together with the potential window and the associated reproducibility statistics.

Normalization is another critical step where uncertainties are often neglected. Geometric-area normalization is straightforward for well-defined RDEs or planar foils, but less so for structured foams, felts, or patterned electrodes, where the true active area is ambiguous. Mass-normalized activity inherits uncertainties from weighing, ink composition, loss during deposition and compositional analysis (*e.g.*, Inductively Coupled Plasma (ICP), X-ray fluorescence (XRF)). ECSA-normalized activity is particularly delicate, because ECSA itself is an effective quantity derived from models that are highly sensitive to porosity, connectivity and local conductivity.

The gold nanoparticle coverage study, mentioned earlier, provides an illustrative example of how this can go wrong.^114^ Over a moderate coverage range, catalytic current scales linearly with total gold surface area, so dividing by that surface gives a meaningful, coverage-independent apparent rate constant. As coverage decreases further, the current–coverage relation bends away from linearity; diffusion layers separate, the voltammetric shape changes, and the apparent rate constant extracted by naively dividing current by total surface becomes strongly coverage dependent. In that regime, surface normalization is no longer an unbiased way to isolate intrinsic kinetics, but a geometric transformation that hides the underlying transition between ensemble and microelectrode behavior.

From an error-analysis standpoint, normalization should therefore be treated as an additional step with its own uncertainty and validity domain. If loading is known only within ±10%, this uncertainty must be propagated into mass-normalized activities. If ECSA is uncertain by ±30%, then ECSA-normalized currents cannot be interpreted more precisely than that, regardless of how smooth the voltammogram appears. Moreover, if several ECSA or surface-determination methods disagree systematically, this spread should be either reflected in error bars or explicitly discussed, rather than hidden, and before dividing current by a given quantity (surface, ECSA, number of sites), it should be checked that current genuinely scales with that quantity over a meaningful range.

In the context of AI-ready data sets, this means that a data set should not just contain best normalized numbers, but also their provenance and uncertainty; how those numbers were normalized, which assumptions were made, and over what range of loading or coverage the underlying linearity was verified.

A natural consequence of rigorous error analysis is that many apparent improvements will not survive quantitative scrutiny. For instance, an alloying strategy may seem to reduce overpotential by 20–30 mV, but within the combined uncertainties of loading, ECSA, film architecture and *iR* correction, the material may be statistically indistinguishable from the benchmark. Similarly, a change in support or synthesis route may initially look promising, but once mass-transport and structural effects are considered, the performance window may overlap completely with that of a simpler reference catalyst. From a scientific perspective, these are valuable negative results. They play the same clarifying role as the careful impurity studies discussed earlier. Therefore, responsible reporting implies that

- Nonimproving modifications and null results are documented, ideally with the same level of quantitative care as positive results, even if only in the Supporting Information.
- Irreproducible literature values are explicitly identified and analyzed when they cannot be reproduced within stated uncertainties, along with plausible reasons (differences in film architecture, porosity, impurities, cell design).
- Curated data sets for modeling and ML include information on variability, failed attempts and dead ends, instead of only best-performing points.

Bringing error analysis and negative results into the foreground ensures that the numbers we report are not only impressive, but also trustworthy, interpretable and genuinely useful for both experimentalists and data-driven approaches in materials electrochemistry.

## 4 Conclusions

The convergence between electrochemistry and materials science has enabled substantial technological progress, but it also demands an evolution in how experiments are designed, interpreted, and reported. A central message emerging from this perspective is that materials used in electrochemistry, particularly at the nanoscale, cannot be treated as well-defined, homogeneous molecular systems. Nanomaterials are intrinsically heterogeneous, structurally complex, and typically evolve under operating conditions. Consequently, the electrochemical response is not an intrinsic property of a nominal material, but rather the result of a specific combination of phase, morphology, structure, and surface properties.

This complexity has important implications for both communities. For electrochemists, it requires moving beyond simplified assumptions inherited from classical models and embracing rigorous nanomaterial characterization. Even seemingly minor factors, such as trace impurities or incidental variations in synthesis, can dominate the observed electrochemical behavior. Moreover, as highlighted throughout this work, the nanomaterial that is electrochemically active is often not the as-synthesized one, but a transformed state that emerges under operating conditions. This underscores the necessity of characterizing nanomaterials not only before, but (possibly) during and definitely after electrochemical measurements, with increasing reliance on *in situ*/*operando* techniques.

For materials scientists, the challenge lies in recognizing that electrochemical measurements are not direct probes of a specific nanomaterial property, but complex responses that convolve kinetics, mass transport, and interfacial phenomena. Parameters such as current density or Tafel slope cannot be interpreted in isolation without careful consideration of surface area, porosity, catalyst loading, and transport limitations. In this context, conventional electrochemical models, developed for idealized systems, must be applied with caution, as their assumptions are often violated in real, nanostructured electrodes.

A recurring theme in this perspective is the critical importance of rigorous methodology and transparent reporting. Careful quantification of uncertainties, systematic error analysis, and detailed description of nanomaterials and experimental conditions are not optional but essential. When such rigor is applied, many apparent performance improvements may fall within experimental uncertainty or arise from uncontrolled variables such as morphology, impurities, or mass transport effects. Correctly reporting improving results is therefore crucial for building a reliable and cumulative knowledge, allowing the field to move forward. These considerations are particularly relevant in the context of data-driven approaches, where the quality of input data directly determines the validity of the conclusions. Data sets that omit variability, uncertainties, or unsuccessful experiments risk reinforcing misleading trends and limiting predictive power. Conversely, well-curated, transparent data sets can significantly accelerate progress by enabling meaningful comparisons and robust model development.

In summary, bridging the gap between electrochemistry and materials science will depend not only on techniques and materials, but also on improved experimental rigor, interdisciplinarity, and a commitment to reproducibility. Only by aligning conceptual frameworks with the true complexity of materials at the nanoscale can we ensure that electrochemical measurements yield insights that are both accurate and transferable.

## Vocabulary

Nanomaterial: substance whose particles are in the size range of 1 to 100 nm. This may have chemical properties different from those of the corresponding bulk material.^153^
Electrocatalysis: detection principle for a chemically modified electrode (CME) in which a charge transfer reaction between a CME and analyte proceeds at a lower overpotential than at the bare electrode. Usually, the effect of electrocatalysis is also an increase of faradaic current.^153^
Overpotential: deviation of the potential of an electrode from its equilibrium value required to cause a given current to flow through the electrode.^153^
Rate-determining step: a rate-controlling (rate-determining or rate-limiting) step in a reaction occurring by a composite reaction sequence is an elementary reaction the rate constant for which exerts a strong effect—stronger than that of any other rate constant—on the overall rate.^153^
Electrode surface area: area of electrode–solution interface. The geometric area, *A*_geom_, is the interfacial area, determined on the assumption that the interface is truly flat (two-dimensional) and calculated using the geometric data of the involved surfaces. The real (true) area, *A*_real_, takes into account nonidealities of the interface (roughness, porosity, *etc.*) and can be measured by a variety of electrochemical methods. The electroactive area is the area calculated from experiments with model electroactive species and may be different from the real surface area in cases where not all of the surface is electrochemically active or accessible.^153^
Electrochemical impedance spectroscopy: electrochemical measurement method of the complex impedance of an electrochemical system as a function of the frequency of a small amplitude (normally) sinusoidal voltage perturbation superimposed on a fixed value of applied potential or on the open circuit potential.^153^
